# Comparison of single and multiple dwell position methods in MammoSite high dose rate (HDR) brachytherapy planning

**DOI:** 10.1120/jacmp.v11i3.3235

**Published:** 2010-05-28

**Authors:** Yongbok Kim, Mark G. Trombetta, Moyed Miften

**Affiliations:** ^1^ Department of Radiation Oncology Allegheny General Hospital Pittsburgh Pennsylvania; ^2^ Drexel University College of Medicine, Allegheny Campus Pittsburgh Pennsylvania; ^3^ Department of Radiation Oncology University of Colorado Denver Aurora Colorado

**Keywords:** single and multiple dwell position methods, interfraction dose variation, MammoSite, HDR brachytherapy

## Abstract

The purpose of this study is to dosimetrically compare two plans generated using single dwell position method (SDPM) and multiple dwell position methods (MDPM) in MammoSite high dose rate (HDR) brachytherapy planning for 19 breast cancer patients. In computed tomography (CT) image‐based HDR planning, a surface optimization technique was used in both methods. Following dosimetric parameters were compared for fraction 1 plans: %PTV_EVAL (planning target volume for plan evaluation) coverage, dose homogeneity index (DHI), dose conformal index (COIN), maximum dose to skin and ipsilateral lung, and breast tissue volume receiving 150% (V150[cc]) and 200% (V200[cc]) of the prescribed dose. In addition, a plan was retrospectively generated for each fraction 2–10 to simulate the clinical situation where the fraction 1 plan was used for fractions 2–10 without modification. In order to create nine derived plans for each method and for each of the 19 patients, the catheter location and contours of target and critical structures were defined on the CT images acquired prior to each fraction 2–10, while using the same dwell‐time distribution as used for fraction 1 (original plan). Interfraction dose variations were evaluated for 19 patients by comparing the derived nine plans (each for fractions 2–10) with the original plan (fraction 1) using the same dosimetric parameters used for fraction 1 plan comparison. For the fraction 1 plan comparison, the MDPM resulted in slightly increased %PTV_EVAL coverage, COIN, V150[cc] and V200[cc] values by an average of 1.2%, 0.025, 0.5 cc and 0.7 cc, respectively, while slightly decreased DHI, maximum skin and ipsilateral lung dose by an average of 0.003, 3.2 cGy and 5.8 cGy, respectively. For the interfraction dose variation comparison, the SDPM resulted in slightly smaller variations in %PTV_EVAL coverage, DHI, maximum skin dose and V150[cc] values by an average of 0.4%, 0.0005, 0.5 cGy and 0.2 cc, respectively, while slightly higher average variations in COIN, maximum ipsilateral lung dose and V200[cc] values by 0.0028, 0.2 cGy and 0.2 cc, respectively. All differences were too small to be clinically significant. Compared to the MDPM, the SDPM combined with a surface optimization technique can generate a clinically comparable fraction 1 treatment plan with a similar interfraction dose variation if a single source is carefully positioned at the center of the balloon catheter.

PACS number: 87.53.Jw

## I. INTRODUCTION

High dose rate (HDR) brachytherapy using a MammoSite balloon applicator (Hologic Corporation, Marlborough, MA) has been widely used in partial breast irradiation for early stage breast cancer patients due to its high reproducibility and stability stemming from the approximate spherical shape of the balloon applicator.^(^ ^1^ ^)^ The lumpectomy cavity easily conforms to the balloon and a single catheter located at the center of the balloon can produce a spherically shaped dose distribution around the balloon. A single HDR brachytherapy source (Ir‐192) can deliver the prescribed dose of 34 Gy to a 1 cm expansion of the lumpectomy cavity with high accuracy by a remotely controlled afterloader in a hypofractionation (10 fractions) scheme. Hence, the MammoSite applicator enables straightforward planning and utilization of a single plan for multi‐fractional delivery. A multiple dwell position method (MDPM) can improve target coverage compared to a single dwell position method (SDPM) by compensating for the inherent anisotropy of the Ir‐192 line source.^(^ ^2^ ^–^ ^4^ ^)^ In addition, the surface optimization technique available in the commercial treatment planning systems (TPS, BrachyVision V6.5, Varian Medical Systems Inc, Charlottesville, VA) can produce a better dose distribution than point dose based techniques (single, six‐ or seven‐point method).^(^ ^4^ ^–^ ^5^ ^)^ MammoSite balloon has been known to be highly reproducible and stable over the HDR treatment fractions when its integrity is visually verified based on two‐dimensional (2D) images, such as X‐ray film or ultrasound images taken before each fraction.^(^ ^6^ ^)^ However, if the shape and location of the MammoSite balloon are evaluated based on three‐dimensional (3D) computed tomography (CT) images acquired prior to each fraction, interfraction variation of the deformity and movement of the balloon to ipsilateral lung and skin could be comprehensively investigated. A recent work has reported that the average variation was clinically insignificant, but clinically significant dose variations (for example, <90% of target coverage and high doses to skin and ipsilateral lung) occurred for some specific fractions. A large variation of balloon eccentricity from a sphere and/or balloon asymmetry resulted in less than 90% of %PTV_EVAL coverage for two fractions out of 188 fractions.^(^ ^7^ ^)^ In general, a MammoSite balloon tightly conformed to the lumpectomy cavity considered as stationary over 10 HDR treatments. Interfraction movements of MammoSite balloon relative to the ipsilateral lung or skin were too small to be visually recognized. However, dosimetric analysis revealed the effects of these small movements on lung and skin maximal dose.^(^ ^7^ ^)^

In this work, a single central dwell position method in MammoSite HDR brachytherapy planning is compared to a multiple dwell position method for 19 breast cancer patients. Both methods utilized a surface optimization technique. Specifically, fraction 1 treatment (original) plans based on CT images were generated using both methods and compared. Furthermore, interfraction dose variations were evaluated for target and critical structures by comparing the derived plan of each fraction 2–10 with the original (fraction 1) plan using several dosimetric parameters.

## II. MATERIALS AND METHODS

### A. Treatment planning

A commercial TPS was employed for CT‐based HDR planning of 19 MammoSite patients. Two plans were generated for fraction 1 (original plan) using SDPM and MDPM. In addition, nine plans (each for fraction 2–10) derived from the original plan were retrospectively generated for each method and each patient. On the axial CT images acquired for fraction 1, the planning target volume (PTV), PTV for plan evaluation purpose (PTV_EVAL) (defined according to the National Surgical Adjuvant Breast and Bowel Project (NSABP) B‐39/ Radiation Therapy Oncology Group (RTOG) 0413 protocol),^(^ ^6^ ^)^ and critical structures such as skin and ipsilateral lung were contoured. The PTV was defined as a 1cm expansion in 3D from the balloon surface excluding the balloon volume itself, and it was a spherical shell with the thickness of 1 cm. In CT image based planning, the volume of balloon was reconstructed from its 2D contours on axial CT images. Because the balloon was inflated with radio‐opaque contrast solution, the balloon could be easily identified using the bone auto‐segmentation tool in the commercial TPS. Additional manual adjustment was made to verify the spherical shape of the balloon by a visual inspection of the 3D view of the balloon. In the retrospective planning for fractions 2–10, the constancy of balloon volume was achieved to ensure that PTV volumes of fractions 2–10 were maintained within 1% variation from that of fraction 1. The PTV_EVAL volume consisted of the PTV, excluding the volumes of skin +0.5 cm and lung/pectoralis muscle. These volumes were automatically delineated by adding 0.5 cm and 1 cm margins to the volume of skin and ipsilateral lung, respectively, in order to remove user dependency in their contouring. In general, the thickness of rib was 5 mm or so, and the thickness of pectoralis muscle from the rib varied over the axial CT slices for a specific patient. Based on the visual inspection of CT images for 19 patients, the typical thickness of pectoralis muscle was about 5 mm from rib, with a range from a few millimeters to a few centimeters. To remove user dependency in contouring the pectoralis muscle over fractions 1–10, in this study, we assumed that the volume of 1 cm expansion from the ipsilateral lung could be considered as the volume of pectoralis muscle. The ipsilateral lung could be easily segmented using the lung auto‐segmentation tool in a commercial TPS and manual adjustment was made, if needed. The volume of ipsilateral breast was defined by adapting the guideline of whole breast irradiation technique in NSABP B39/RTOG 0413 protocol.^(^ ^6^ ^)^ First, a 3D volume of a box was constructed to include whole ipsilateral breast from midsternum (medial boundary) to midaxillary line (lateral boundary) in axial CT slices, which covered from sternal manubrium joint (cephalad boundary) to 1–2 cm below the inframammary line (caudal boundary). The ipsilateral breast was defined by subtracting volume of non‐patient body (outside of patient), pectoralis muscle, chest wall and balloon from the 3D box. To reduce the uncertainty in localizing the catheter lumen inside the MammoSite balloon, the CT images were rotated in 3D and their intensity settings (window/level) were modified to best visualize the lumen inside the balloon. Depending upon the volume of balloon, dose points ranging from 3000 to 3500 were generated on the surface of the balloon +1 cm (1 cm expansion of the balloon in 3D). The desired fractional dose of 3.4 Gy was prescribed to these dose points.^(^ ^4^ ^–^ ^5^ ^)^ The dose was delivered twice daily over five consecutive days (i.e. 10 fractions) with the total delivered dose of 34 Gy. At our institution, the goal of target coverage is more than 90% of the PTV_EVAL volume to be covered with the prescribed dose (V100%>90%).

**Figure 1:**
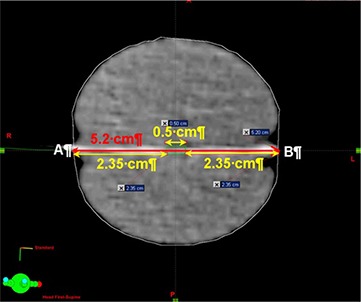
The verification of placement of a single source at the center of balloon for fraction 1 plan of patient 10. The first and last source positions define the single source position. The balloon diameter ($D_{\text{balloon}}$) is 5.2 cm. The first source position is 2.35 cm from the tip of catheter and the last source position is 2.85 cm (2.35cm+0.5 cm). The same measurement as 2.35 cm from both ends of the source to points A and B (intersection points between catheter and balloon) confirms positioning accuracy.

A treatment plan was generated using the MDPM in conjunction with a surface optimization technique and used for the fraction 1 treatment. In the MDPM planning, the possible multiple dwell positions were determined by the most proximal and distal position of balloon catheter in the source position parameter module of TPS. For instance, the balloon with a radius of 5.2 cm in Fig. 1 had the most distal position of 0.5 cm back from the tip of catheter, which was a safety margin to avoid hitting the tip of catheter during treatment. The most proximal position was 5 cm back from the tip of catheter. The number of dwell positions could be calculated by the following equation.

(1) $$N_{\text{Dwell}}=\frac{\left(\text{Proximal}\;\text{position})-(\text{Distal}\;\text{position}\right)}{\left(\text{Size}\;\text{of}\;\text{source}\;\text{step}\right)}$$

The source step size used was 0.5 cm and the number of possible dwell positions was nine from the calculation of (5 cm−0.5 cm)/0.5 cm. The fraction 1 plan was also adopted for fraction 2–10 treatments without any modification unless a significant change of the MammoSite applicator was observed, such as >2 mm deviation of balloon diameter, large increase of air gap volume which makes %PTV_EVAL coverage <90%, or rupture of balloon. In order to simulate this clinical situation, the dose distribution of the fraction 1 plan was superimposed on the anatomy and the catheter location available on CT images from each fraction. The dwell time distribution of the fraction 1 plan (original plan) was manually transferred to the dwell positions defined on the CT scans for fractions 2–10 to derive 9 plans (each for fractions 2–10) for each patient.

An additional plan was retrospectively generated for fraction 1 using the SDPM along with a surface optimization technique. Following the same approach as in MDPM, nine derived plans (each for fractions 2‐10) were simulated by manually transferring the single dwell time of the fraction 1 plan (original plan) to the central dwell position identified for fractions 2–10. To improve the accuracy in locating a single dwell position at the center of the balloon, the 3D CT images matrix were rotated and a CT slice showing the largest diameter of balloon was selected (Fig. 1). In general, the CT slice was not necessarily aligned with the CT image scan plane. After the brightness/contrast of the CT image was adjusted to best depict the balloon catheter, the single dwell position was identified using Eq.1 with $N_{\text{Dwell}}$ of 1. The distal position was determined by the magnitude of “$(D_{\text{balloon}}-0.05\;\text{cm})/2$” back from the tip of the catheter, where the $D_{\text{balloon}}$ is the balloon diameter along the catheter (Fig. 1). The proximal position was defined by the distal position plus the source step size of 5 mm such as “$0.5\;\text{cm}+(D_{\text{balloon}}-0.05\;\text{cm})/2$” back from the tip of catheter. The two measurements shown in Fig. 1 from both ends of the source to the intersections (points A and B) between catheter and balloon validate the accuracy of positioning the single source.

### B. Dosimetric parameters

A number of dosimetric parameters were used for plan comparison such as %PTV_EVAL coverage, dose homogeneity index (DHI), dose conformal index (COIN), maximum dose to the skin and ipsilateral lung, as well as breast tissue volume receiving 150% and 200% of the prescribed dose (V150[cc] and V200[cc]). For the target volume, the dose coverage of %PTV_EVAL was calculated as the percentage volume of PTV_EVAL receiving the prescribed dose (V100%) after accounting for the trapped air gap on the surface of balloon, if present.^(^ ^6^ ^)^ In addition, DHI^(^ ^7^ ^–^ ^8^ ^)^ and COIN^(^ ^7^ ^,^ ^9^ ^–^ ^10^ ^)^ values were evaluated over the PTV_EVAL volume. The DHI value was computed by DHI=(V100[cc]−V150[cc])/V100[cc]. The COIN value was calculated by $\text{COIN}=c_{1}\times c_{2}$, where $c_{1}=\text{PTV}\_{\text{EVAL}}_{\text{ref}}/\text{PTV}\_\text{EVAL}$ and $c_{2}=\text{PTV}\_{\text{EVAL}}_{\text{ref}}/V_{\text{ref}}$. $\text{PTV}\_{\text{EVAL}}_{\text{ref}}$ is defined as the volume of the PTV_EVAL which is enclosed by at least the prescribed dose and $V_{\text{ref}}$ is the tissue volume that is enclosed by the prescribed isodose line. In addition, the maximum dose to the skin and ipsilateral lung^(^ ^7^ ^)^ was assessed. To reduce the incidence of fat necrosis^(^ ^6^ ^,^ ^11^ ^)^ and dose inhomogeneity in the target, the NSABP B39/RTOG 0413 protocol limited the volume of breast tissue receiving excessive doses (V150[cc] <50 cc and V200[cc] <10 cc).^(^ ^6^ ^)^ Hence, the V150[cc] and V200[cc] values were evaluated. Furthermore, the same metrics were utilized to investigate the interfraction dose variations by comparing the plan of each fraction 2–10 with the fraction 1 plan for both SDPM and MDPM.

## III. RESULTS

### A. Dosimetric comparison for fraction 1 plans

**Figure 2:**
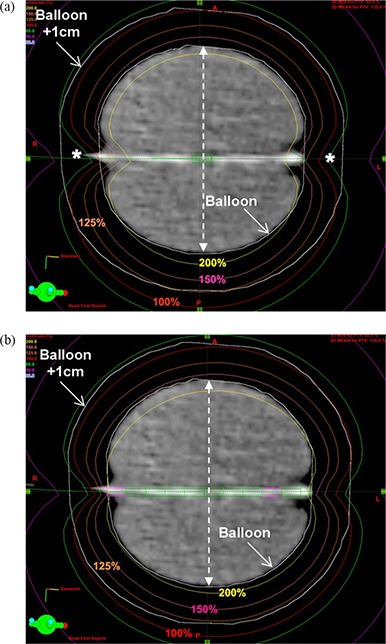
Comparison of two‐dimensional dose distribution on the plane showing maximum balloon diameter between two plans using (a) SDPM and (b) MDPM for fraction 1 plan of patient 10.

The dosimetric parameters of fraction 1 plans for 19 patients are summarized in Table 1 with mean, standard deviation, minimum and maximum values. Since the comparison was performed between two plans in the same individuals and the measurement data could not be guaranteed from a normally distributed population, a non‐parametric test (Wilcoxon matched‐pairs signed‐ranks test) was used to analyze the statistical difference.^(^ ^12^ ^)^ On average, the MDPM resulted in slightly higher %PTV_EVAL coverage and COIN value by 1.2% and 0.025, respectively, as compared to the SDPM. On the other hand, the DHI, maximum skin dose and maximum ipsilateral lung dose values were slightly lower by an average of 0.003, 3.2 cGy and 5.8 cGy, respectively. The volume of breast tissue receiving high doses was slightly increased with MDPM by an average of 0.5 cc for V150[cc] and 0.7 cc for V200[cc] in comparison with the SDPM. The improvement in %PTV_EVAL coverage by the MDPM is primarily the result of compensating for the source anisotropy which causes under dosage (cold spot denoted as asterisks in Fig. 2(a)) at the polar regions along the catheter axis of the MammoSite balloon applicator.^(^ ^2^ ^)^ Figure 2 compares dose distributions in a CT slice between the SDPM and MDPM. The asymmetry of the balloon catheter in the equatorial axis (direction of dashed line with two end arrows perpendicular to the catheter axis in Fig. 2) cannot be corrected even with the MDPM (Fig. 2(b)). Although the differences were statistically significant (p‐value <0.05 in Table 1) for most of parameters (except for the DHI and V150[cc] values), they were too small to be considered clinically significant. On average, the percent (%) difference calculated by $({\text{Value}}_{\text{SDPM}}-{\text{Value}}_{\text{MDPM}})/{\text{Value}}_{\text{MDPM}}\times 100$ was less than 3% for all parameters except for V200[cc]. Despite the large average percent difference (−12.4%) of V200[cc] value, the absolute difference was less than 1 cc (−0.6 cc). Hence, the SDPM can produce a comparable treatment plan to the MDPM for fraction 1.

**Table 1:** Dosimetric comparison of the single and multiple dwell position methods (SDPM and MDPM) for fraction 1 treatment plans of 19 patients.

	*SDPM*				*MDPM*				*p‐value*
	*Mean*	*SD*	*Min*	*Max*	*Mean*	*SD*	*Min*	*Max*	
%PTV_EVAL coverage [%]	94.4	2.1	90.4	97.2	95.6	1.8	91.9	97.8	0.0204
Dose homogeneity index (DHI)	0.647	0.037	0.565	0.707	0.644	0.039	0.561	0.706	0.4180
Dose conformal index (COIN)	0.821	0.072	0.692	0.930	0.846	0.075	0.703	0.929	<0.0001
Maximum skin dose [cGy]	321.0	103.9	135.0	610.1	317.8	115.5	134.0	683.4	0.0095
Maximum ipsilateral lung dose [cGy]	207.8	122.7	53.1	474.1	202.0	117.8	51.7	444.6	0.0124
V150[cc]	30.2	2.7	26.2	35.8	30.7	2.3	27.4	36.0	0.1819
V200[cc]	4.1	1.9	0.5	8.4	4.8	2.1	0.9	8.1	0.0005

SD=standard deviation; Min=minimum value; Max=maximum value; V150[cc]=absolute volume of breast tissue receiving 150% of the prescribed dose; V200[cc]=absolute volume of breast tissue receiving 200% of the prescribed dose; p‐value was calculated by Wilcoxon matched‐pairs signed‐ranks test (non‐parametric test).

### B. Comparison of interfraction dose variation

The interfraction dose variations of the fraction 2–10 plans were evaluated relative to the fraction 1 plan for both methods. The total number of data sets used was 169 excluding two missing CT datasets. The independent 169 interfraction dose variation data were acquired for each method and summarized in Table 2 with mean and standard deviation values. As they were not drawn from a normally distributed population, Mann‐Whitney rank sum test (nonparametric test) was used to analyze the statistical difference between the two methods.^(^ ^12^ ^)^ The SDPM resulted in slightly smaller interfraction variations in %PTV_EVAL coverage, DHI value, maximum skin dose, and V150[cc] value by an average of 0.4%, 0.0005, 0.5 cGy, and 0.2 cc, respectively, compared to the MDPM. In contrast, it slightly increased the interfraction variations in COIN value, maximum ipsilateral lung dose, and V200[cc] value by an average of 0.0028, 0.2 cGy, and 0.2 cc, respectively. However, all the differences are clinically and statistically insignificant (p‐value >0.05).

**Table 2:** Comparison of interfraction dose variations for each fraction 2–10 relative to fraction 1 (169 data points for 19 patients) using the single and multiple dwell position methods (SDPM and MDPM).

	*SDPM*		*MDPM*		*p‐value*
	*Mean*	*SD*	*Mean*	*SD*	
%PTV_EVAL coverage [%]	0.0	2.0	−0.4	2.6	0.2398
Dose homogeneity index (DHI)	0.0017	0.0198	−0.0022	0.0155	0.0829
Dose conformal index (COIN)	0.0057	0.0317	−0.0029	0.0416	0.0788
Maximum skin dose [cGy]	7.7	44.7	8.2	47.6	0.7435
Maximum ipsilateral lung dose [cGy]	−16.3	30.2	−16.1	29.3	0.9273
V150[cc]	0.05	1.73	0.25	1.26	0.3440
V200[cc]	−0.23	1.24	−0.03	1.08	0.1264

SD=standard deviation; V150[cc]=absolute volume of breast tissue receiving 150% of the prescribed dose; V200[cc]=absolute volume of breast tissue receiving 200% of the prescribed dose; p‐value was calculated by Mann‐Whitney rank sum test (non‐parametric test).

### C. Positioning error of the single source in the SDPM

**Figure 3:**
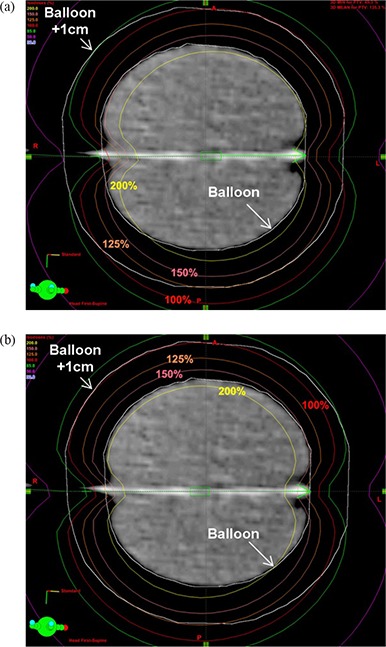
Shift of the dose distributions (from Fig. 2(a)) for fraction 1 SDPM plan of patient 10 due to the error in positioning a single source by 2 mm distally (a) and proximally (b).

When the SDPM was employed, attention was paid to the accurate positioning of the single source at the center of balloon catheter. If there is any significant error in positioning the single source, the resultant dose distribution may be different in some degree from the plan without error (reference plan). To illustrate this, a 2 mm positioning error was simulated for the fraction 1 plan of patient 10. The single source position at center of the balloon catheter was forced to shift both distally and proximally by 2 mm. The modified plans were compared to the reference plan in terms of dose distribution in Fig. 3 and dosimetric parameters in Table 3. The shifted plan was degraded from the reference plan for % PTV_EVAL coverage by 2.0%, DHI by 1.3%, and COIN by 3.9%, on average, relative to the reference plan calculated by $({\text{Value}}_{\text{Shift}}-{\text{Value}}_{\text{Reference}})/{\text{Value}}_{\text{Reference}}\times 100$. The average deviation of maximum skin and ipsilateral lung dose was 15.5 cGy (5.7%) and 5.1 cGy (4.9%), respectively. The breast tissue volume receiving high doses was increased by an average of 0.6 cc (1.6%) for V150[cc] and 1.0 cc (26.9%) for V200[cc], respectively.

**Table 3:** Deviation of dosimetric parameters from a reference plan (fraction 1 SDPM plan of patient 10) to two plans by shifting a single dwell position by 2 mm in the distal and proximal directions.

	*Reference*	*2 mm shift distally*	*2 mm shift proximally*
%PTV_EVAL coverage [%]	92.9	90.6	91.5
Dose homogeneity index (DHI)	0.6984	0.6869	0.6920
Dose conformal index (COIN)	0.8061	0.7668	0.7818
Maximum skin dose [cGy]	268.7	253.5	284.4
Maximum ipsilateral lung dose [cGy]	104.1	109.4	99.2
V150[cc]	36.0	36.9	36.3
V200[cc]	3.7	4.8	4.5

V150[cc]=absolute volume of breast tissue receiving 150% of the prescribed dose; V200[cc]=absolute volume of breast tissue receiving 200% of the prescribed dose.

## IV. DISCUSSION

While several authors reported an average of 76%,^(^ ^13^ ^)^ 77.6%^(^ ^3^ ^)^ and 85%^(^ ^4^ ^)^ target coverage (V100%) by using a SDPM in conjunction with a single point optimization method, the results in this study showed that the SDPM yielded more than 90% of %PTV_EVAL coverage for all 19 patients. Such a high %PTV_EVAL coverage with an average of 94.4% (only 1.2% lower than the MDPM) was achieved primarily due to the accurate placement of the single dwell position at the center of the MammoSite balloon catheter. In addition, utilization of the surface optimization technique can improve target coverage.^(^ ^4^ ^)^ The method of prescription in this study was different from that used in the early MammoSite treatments. As Edmundson et al.^(^ ^1^ ^)^ described, the prescription in the early MammoSite treatments was originally to a single point of 1 cm expansion from the balloon surface on the equatorial axis. Dickler et al.^(^ ^3^ ^)^ and Kirk et al.^(^ ^4^ ^)^ showed that for the same single prescription point the MDPM was able to achieve better target coverage compared to the SDPM. In this study, the dose of 3.4 Gy was prescribed to each of the several thousand dose points at the surface of the 1 cm expansion of the balloon in 3D.^(^ ^5^ ^)^ This surface optimization technique improved %PTV_EVAL coverage despite the use of SDPM. In clinical practice, the positioning error of the source at the center of the spherical balloon is probably less of an issue compared to the source anisotropy effect because it is not difficult to position the source within 1 mm accuracy.

This study demonstrates that an MDPM plan is slightly better than a SDPM plan for dose conformality to the target and for sparing normal tissues. The enhanced dose shaping capability using multiple dwell positions can result in less target dose homogeneity.^(^ ^4^ ^)^ Therefore, compared to the SDPM, the MDPM can produce a plan which is slightly improved in dose conformality while it may be more vulnerable to interfraction dose variations. The NSABP B39/RTOG 0413 protocol recommended 2D X‐ray film or ultrasound images to verify the integrity of MammoSite balloon prior to each fraction.^(^ ^6^ ^)^ In this study, nine additional CT scans were used for verification purpose as well as retrospective planning. The additional radiation dose to whole breasts from those nine CT scans were 0.15 Gy, based on the data from Brenner and Hall.^(^ ^14^ ^)^ The dose is insignificant compared to a prescription dose of 34 Gy, and the potential risk associated with the additional dose from nine CT scans may be not large for elderly patients. However, the increasing exposure to young patients may increase the risk of secondary cancer in the future.

## V. CONCLUSIONS

Compared to the multiple dwell position method, the single dwell position method combined with a surface optimization technique can generate a clinically comparable plan for fraction 1. Furthermore, both methods show similar interfraction dose variation. However, when the single dwell position method is utilized, positioning the single dwell position at the center of balloon catheter is critical.
